# Synthesis, Thermogravimetric Analysis, and Kinetic Study of Poly-*N*-Isopropylacrylamide with Varied Initiator Content

**DOI:** 10.3390/polym15112427

**Published:** 2023-05-23

**Authors:** Agnieszka Gola, Tomasz Knysak, Igor Mucha, Witold Musiał

**Affiliations:** 1 Department of Physical Chemistry and Biophysics, Pharmaceutical Faculty, Wroclaw Medical University, Borowska 211a, 50-556 Wrocław, Poland; agnieszka.gola@umw.edu.pl (A.G.); tomekknysak06@interia.eu (T.K.); 2 Department of Basic Chemical Sciences, Faculty of Pharmacy, Wroclaw Medical University, Borowska 211a, 50-556 Wrocław, Poland; igor.mucha@umw.edu.pl

**Keywords:** *N*-isopropylacrylamide, TGA/DTG, non-isothermal, activation energy

## Abstract

The thermal decomposition and kinetic parameters of four polymers, PN-1, PN-05, PN-01, and PN-005, were determined by thermogravimetry (TGA/DTG) under non-isothermal conditions. *N*-isopropylacrylamide (NIPA)-based polymers were synthesized by the surfactant-free precipitation polymerization (SFPP) with different concentrations of the anionic initiator potassium persulphate (KPS). Thermogravimetric experiments were carried out in the temperature range of 25–700 °C at four heating rates, 5, 10, 15, and 20 °C min^−1^, under a nitrogen atmosphere. Poly NIPA (PNIPA) showed three stages of mass loss during the degradation process. The thermal stability of the test material was determined. Activation energy values were estimated using Ozawa, Kissinger, Flynn–Wall–Ozawa (FWO), Kissinger–Akahira–Sunose (KAS), and Friedman (FD) methods.

## 1. Introduction

Drug design is a very complex process involving many steps and studies, as well as high risk and uncertainty [1]. One of these stages is drug stability testing. This is conducted to determine the stability, durability, and usability of the drug and to ensure that it is safe over a certain period of time, as well as to test the effects of selected factors on the evaluated molecule. The factors influencing the type of changes that occur can be manufacturing-related or external. The stability of the active pharmaceutical ingredient (API) is one of the key elements determining the quality and therapeutic efficacy of the drug and its safety for the patient. Currently, standard drug delivery methods face the problem of drug distribution throughout the body. One way to solve this problem is to use drug carriers to deliver the drug to its proper site of action. The benefits of using this method include reduced drug dosage minimized toxic effects on healthy tissues, the additional protection of the delivered substances from environmental factors, and increased bioavailability. Presently, there is a great demand for research into new drug delivery systems or the modification of existing systems. Among other things, a well-designed drug carrier should have a controlled release rate and be non-toxic, biodegradable, and physically and chemically stable. Therefore, determining the stability of drug carriers is an extremely important aspect of manufacturing, as is determining the stability of the APIs.

Among the many drug carriers used, polymeric drug carriers are the most popular. Polymeric carriers can be synthetic (polyethylene glycol—PEG, polylactic acid—PLA, poly *N*-isopropylacrylamide—PNIPA, poly *N*-vinylcaprolactam—PNVCL) [2,3,4,5] or natural (chitosan, hialurionian, cellulose, and starch) [6,7,8,9]. Modern materials used as drug carriers should be characterized by unique externally spacetime-controlled properties in a physiological environment, allowing precise drug delivery to the target site and tuned release at the same time. In this case, ideal candidates, due to their reversible sensitivity to environmental stimuli, such as temperature, pH, UV radiation, or the concentration of a specific metabolite in the human body, are the so-called smart polymers [10,11,12,13,14].

Both hydrophobic isopropyl and hydrophilic amide groups, present in the network structure of the poly *N*-isopropyl acrylamide (PNIPA), provide property specific to PNIPA-reversible sensitivity to external temperature stimuli. Due to these characteristic features, PNIPA is included in the group of so-called smart polymers. In an aqueous environment, thermosensitive PNIPA exhibits a unique lower critical solubility temperature (LCST) of ~32 °C, close to the physiological temperature of the human body [15,16,17,18]. The phase transition temperature, as an important parameter, can determine the properties of the polymer in the biological phase. Due to the ease of modification and precise in vivo and in vitro control of such a parameter as temperature, together with a unified phase transition after changing the external temperature, PNIPA is of great interest in biomedical applications as an intelligent drug carrier [19,20]. Substances classified as potential drug carriers must be characterized by specific physicochemical properties, i.e., particle size, particle surface charge, LCST, and thermal stability, and can meet a number of criteria, such as demonstrating the high binding efficiency of the active substance, a controlled release rate, and maintaining drug activity during transport and storage.

State-of-the-art stability assessment methods allow the rapid evaluation of the stability of drug products. Analytical techniques with strong research potential to support the quality assessment and confirmation of the identity, stability, and purity of APIs, excipients, and drug carriers include, but are not limited to, the methods used in this research work. These include X-ray powder diffraction (XRPD), Fourier transform infrared spectroscopy–attenuated total reflectance (ATR-FTIR), and thermogravimetric analysis (TG), which were widely applied in the evaluation of methylcellulose (ME), polyacrylic acid (PA), chitosan, and PNIPA, among others [21,22,23,24,25,26].

The fast and non-destructive XRPD method made it possible to provide information about the crystallographic structure and detect morphological changes or crystallographic impurities [27].

An interpretation of ATR-FTIR spectra allows the presence or absence of specific functional groups in the characteristic spectral region in the 4000–200 cm^−1^ range to be determined, as well as the chemical structure of the compounds under study [28,29].

TG analysis was used to determine the purity and stability of drug substances, excipients, and drug carriers, as well as in physicochemical studies to determine the thermodynamic and kinetic parameters of reactions [30,31,32,33].

The kinetics model was based on the basic kinetic Equation (1), derived from the Arrhenius relation of the transformation of the degree of mass loss (*α*) for thermal degradation, where *f*(*α*) is the function that determines the course of the reaction, *k*(*T)* is the reaction rate constant, and *t* is time.

(1) $$\frac{d\alpha }{dt}=k\left(T\right)f\left(\alpha \right),$$

To perform the kinetic analysis of the experimental data and obtain kinetic parameters for thermal degradation, we used five isoconversional methods. The activation energy (*E_a_*) and pre-exponential factor (*A*) were obtained by four non-isothermal methods described by Ozawa [34], Kissinger [35], Flynne–Wall–Ozawa (FWO) [34,36], Kissinger–Akahira–Sunose (KAS) [35,37] and one isothermal method proposed by Friedman (FD) [38], respectively.

According to the Ozawa and Kissinger methods, the activation energy and pre-exponential factor of a solid-state reaction were determined based on the relationship between the temperature of the maximum mass loss rate *T_m_* and the heating rate *β* (°C·min^−1^). The kinetic parameters obtained by Ozawa and Kissinger methods were calculated using Equations (2) and (3), respectively:

(2) $$\ln\beta =\ln\left(\frac{AE_{a}}{R}\right)-\frac{E_{a}}{RT_{m}},$$

(3) $$\ln\frac{\beta }{T^{2}}=\ln\left(\frac{AR}{E_{a}}\right)-\frac{E_{a}}{RT_{m}},$$

Plotting ln(*β/T*^2^) versus 1/*T* K^−1^ and ln*β* versus 1/*T* K^−1^ yielded a straight line. Thus the activation energy *E_a_* and pre-exponential factor could be derived from the slope and intercept of the plotted regression lines. The Ozawa and Kissinger methods were derived from the Johnson–Mehl–Avrani theory [39].

The FWO and KAS methods are based on measuring the temperature corresponding to set values of the conversion degrees (*α*) in experiments with different heating rates (*β*). The kinetic equations for the FWO and KAS methods were, the results of Doyle’s [40] and Coast–Redfren [41] approximations for integral, respectively, and are most commonly presented as Equation (4) for FWO and Equation (5) for KAS, where *g*(*α*) is the integral conversion [31].

(4) $$\ln\left(\beta_{i}\right)=\ln\left(\frac{A_{\alpha }\cdot E_{a_{\alpha }}}{R\cdot g(\alpha )}\right)-5.331-1.052\frac{E_{a_{\alpha }}}{R\cdot T_{\alpha_{i}}},$$

(5) $$\ln\left(\frac{\beta_{i}}{T_{\alpha_{i}}^{2}}\right)=\ln\left(\frac{A_{\alpha }\cdot R}{E_{\alpha }\cdot g(\alpha )}\right)-\frac{E_{a_{\alpha }}}{R\cdot T_{\alpha_{i}}},$$

The plot on the left side of Equations (4) and (5) versus the inverse of *T_α_*. for the conversion constant (*α*) and at different heating rates represents a linear function. The apparent activation energy could be evaluated directly from the slopes −1.052·*E_α_/R* and *E_α_/R* by means of the FWO and KAS methods, respectively. The pre-exponential factor could be obtained from the cut-off from the ordinate axis.

The Friedman (FD) method is the most common differential and isothermal method based on a comparison of mass loss rates (*dα/dt*) for conversion degrees (*α*) and relies on Equation (6). In its logarithmic form, where *f*(*α*) is the conversion function:

(6) $$\ln\left(\beta \frac{d\alpha }{dt}\right)≅\ln\left(\beta \frac{d\alpha }{dT}\right)=\ln(A\cdot f(\alpha ))-\frac{E_{a}}{R\cdot T_{\alpha }},$$

The FD method does not include any mathematical approximations; therefore, it is more accurate than the FWO and KAS methods. The values of the apparent activation energy *E_a_* and the pre-exponential factor *A* could be calculated from the regression of each straight line obtained from a linear plot of ln(*β·dα*/*dT*) with 1/*T* over a wide range of conversion measurements with different heating rates.

To the best of our knowledge, there are works on the thermal studies and thermal degradation kinetics of NIPA and derivatives [42,43,44,45,46]. However, our achievement is the first to present an analysis and comparison of the kinetic parameters obtained from thermogravimetric studies of four NIPA polymers synthesized with different amounts of initiator and to study the effect of initiator concentration on these parameters.

The aim of this work was to synthesize four *N*-isopropylacrylamide polymers PN-1, PN-05, PN-01 and PN-005, and study the thermal degradation of the synthesized polymers by thermogravimetric analysis, to determine the kinetic parameters (*E_a_*, *A*) of the thermal degradation process using various calculations procedures (Kissinger, Ozawa, KAS, FWO, FD). Moreover, the evaluation of the effect of the initiator level on the selected thermal and kinetic parameters, which is key to the determination of the thermal behavior of the studied thermosensitive polymers with different hydrodynamic diameters, was performed.

## 2. Materials and Methods

### 2.1. Materials

*N*-isopropylacrylamide (NIPA, 99%, Sigma-Aldrich Chemical, St. Louis, MO, USA), potassium persulfate (KPS, 98%, BDH Laboratory Suppliers (GPR^TM^). All chemicals were used as received without further purification. The dialysis tubing cellulose membrane of molecular mass cut-off MWCO: 12,000–14,000 Da were obtained from Sigma-Aldrich Chemical, St. Louis, MO, USA. Deionized water with a conductivity of 0.06 μS·cm^−1^ was taken from a microfiltration capsule 0.22 μm in a HLP 20 system (Hydrolab, Straszyn, Poland), which fulfilled requirements of PN-EN ISO 3696:1999 for analytical laboratories.

### 2.2. Synthesis of PNIPA Nanoparticles

Thermosensitive PNIPA nanoparticles were prepared by free radical precipitation polymerization without an emulsifier (surfactant-free precipitation polymerization, SFPP). An appropriate amount (0.300–5.973 g) of the dry weight initiator—the KPS sample—was added in a four-necked round bottom flask filled with 900 mL deionized water and heated to 70 °C. Then, 5 g (with an accuracy of 0.006 g) of the monomer—NIPA dissolved in 100 mL of deionized water—was added to the reaction vessel. Table 1 shows the used initial amounts of the reactants. The reaction flask was equipped with an Allihn reflux condenser, Teflon magnetic stirrer, a nitrogen inlet and a temperature sensor. Polymerization was carried out for 6 h at 70 °C under a stream of nitrogen. The reaction mixture was stirred at 250 rpm. The final products were purified by dialysis for 5 days and recovered by liofilization.

### 2.3. Powder X-ray Diffraction Analysis (PXRD)

PXRD data of the powders were recorded on a Bruker D2 PHASER diffractometer (Bruker AXS, Karlsruhe, Germany) with a LynxEYE detector using a Cu Kα1.2 radiation source (λ = 1.5418 Å). All samples were measured at 295 K. Data were collected in the Bragg–Brentano (θ/2θ) horizontal geometry ranging from 5° to 70° (2θ). A step size of 0.02° (2θ) at 1.0 sec/step was used. The optics of the D2 PHASER diffractometer consisted of a 2.0° Soller slit module, a 0.6 mm shutter and a Ni filter. The X-ray tube operated at 30 kV and 10 mA. XRPD patterns were processed using the Diffrac.Eva V 3.2 software, (Bruker AXS, Karlsruhe, Germany).

### 2.4. Fourier Transform Infrared Spectroscopy (FTIR)

FTIR-ATR spectra were collected using a Nicolet iS50 FT-IR spectrometer (Thermo Fisher Scientific, Madison, WI, USA) equipped with a diamond crystal attenuated total reflectance (ATR) sampler accessory and DLATGS detector (Deuterated L-alanine doped Triglycine Sulphate) with a KBr window over the scanning range of 400–4000 cm^−1^. Each spectrum comprised 32 co-added scans recorded at a spectral resolution of 4 cm^−1^. Background readings were collected and subtracted from each spectrum before the data output. Infrared data acquisition and the elemental analysis of the results were performed using Thermo Scientific OMNIC spectroscopy software (Version 9, Thermo Fisher Scientific, Madison, WI, USA). Measurements were made by pressing a micro portion of the sample on the diamond against the anvil of the Golden Gate ATR unit.

### 2.5. Thermogravimetric Analysis (TGA)

Thermogravimetric analyses were carried out on a TG 209 F1 Libra instrument (Erich NETZSCH GmbH & Co. Holding KG, Selb, Germany) under non-isothermal conditions. Samples (5 ± 0.03 mg) were placed in 150 mL aluminum crucibles and heated between 25 °C and 700 °C to determine the thermal stability of the samples. Experiments were conducted at different heating rates for 5, 10, 15 and 20 °C min^−1^ in a nitrogen environment (flow rate of 25 mL·min^−1^). The instrument was equipped with a unique BeFlat function which compensated for any external factors influencing the measurements and precluded the need to perform the baseline. Measurements, evaluating the results and analysis of the obtained data were carried out using Netzsch Proteus 7.1.0 analysis software (Selb, Germany).

## 3. Results

### 3.1. Synthesis

The synthesis yielded four polymers: PN-1, PN-05, PN-01 and PN-005. Each synthesis was carried out under the same conditions and used identical amounts of monomer and decreasing amounts of the initiator, potassium persulphate (KPS) per liter of the mixture. A summary of the composition to prepare four poly-NIPA nanoparticles is shown in Table 1. The numbers in the polymer names indicate the number of moles for the radicals used in the synthesis per mole of the monomer.

**Scheme 1:**
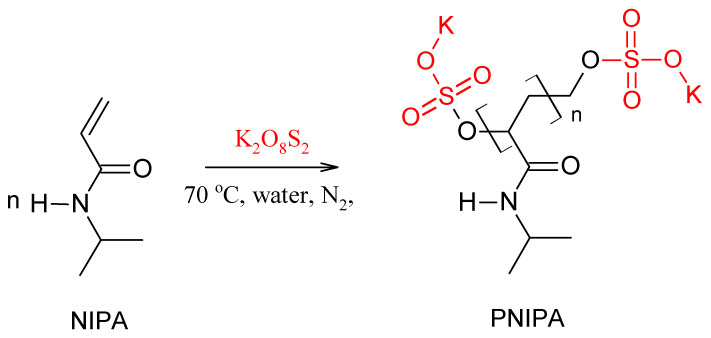
Exemplary scheme of radical polymerization reaction of NIPA in water at 70 °C activated by K_2_O_8_S_2_.

An example schematic diagram illustrating a possible version of the radical polymerization of NIPA by radicals derived from the decomposition of potassium persulfate in water at 70 °C is shown in Scheme 1.

### 3.2. Powder X-ray Diffraction Analysis (XRPD)

**Figure 1:**
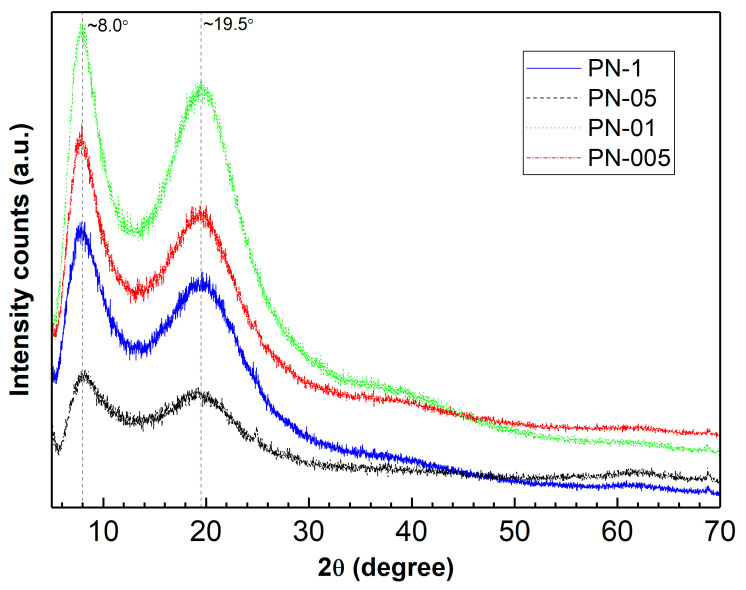
Powder X-ray diffraction pattern (PXRD) of synthetized polymers PN−1, PN−05, PN−01, PN−005.

The lyophilized and powdered samples of synthesized polymers were characterized by XRPD measurements. XRPD patterns of the synthesized pNIPA homopolymer powder are shown in Figure 1. Diffractograms of the polymers show two reflection maxima, one broad diffraction peak at a diffraction angle of about 20° and a second less intense peak at a diffraction angle of about 8°, what is consistent with the published data [48,49].

### 3.3. Attenuated Total Reflection Fourier-Transformed Infrared Spectroscopy (ATR-FTIR)

**Figure 2:**
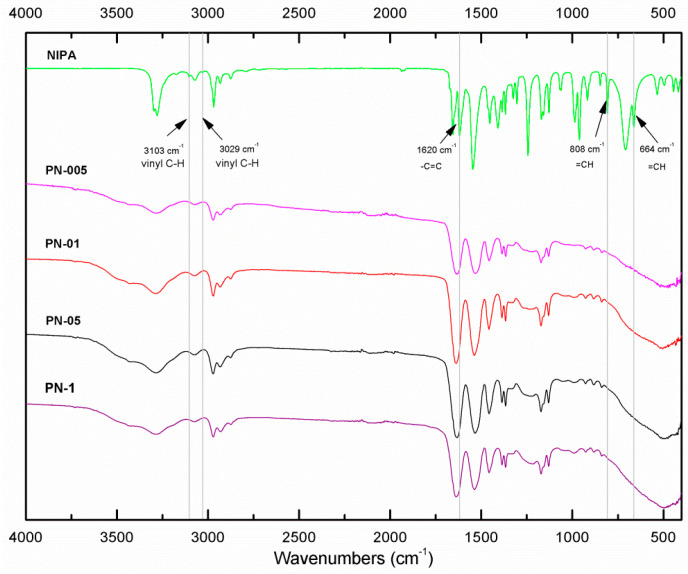
Fourier-transformed infrared spectroscopy with attenuated total reflectance (ATR-FTIR) of monomer *N*-isopropylacrylamide and four synthesized polymers PN-1, PN-05, PN-01, PN-005.

A comparison of the ATR-FTIR spectra of the NIPA monomer and prepared NIPA polymers, PN-1, PN-05, PN-01 and PN-005, in the range of 4000–400 cm^−1^ are shown in Figure 2. Characteristic peaks at 3103 and 3029 cm^−1^ could be attributed to the stretching vibrations of the unsaturated C=C bond [49,50,51], which could be found in the NIPA monomer. In addition, the NIPA spectrum showed characteristic peaks at 1620 cm^−1^ (C=C stretching vibrations) [52,53] 808 cm^−1^ (=C-H deformation out of plane) and 664 cm^−1^ (=C-H wagging vibrations) [50,54]. 

### 3.4. Thermogravimetric Analysis (TGA)

**Figure 3:**
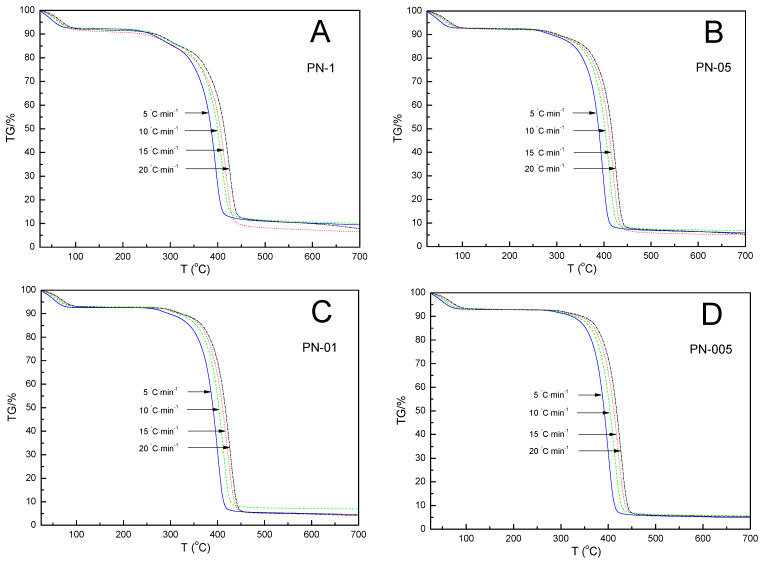
Mass loss (TG%) vs. temperature of polymers, PN-1 (**A**), PN-05 (**B**), PN-01 (**C**), PN-005 (**D**), obtained at different heating rates at 5, 10, 15 and 20 °C min^−1^ in a nitrogen atmosphere of 25 mL·min^−1^.

**Figure 4:**
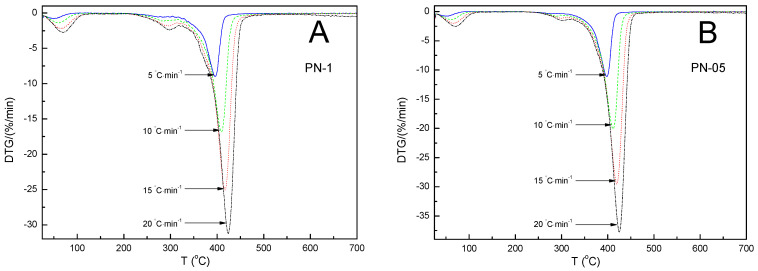

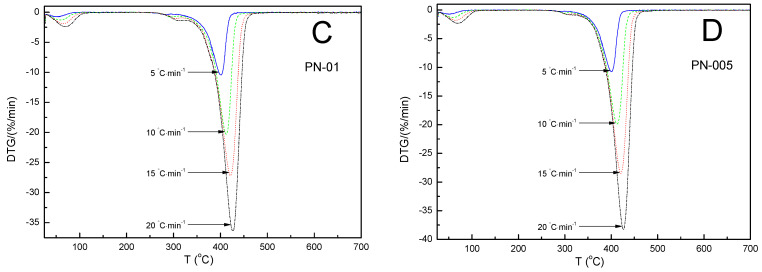
Derivative mass loss (DTG %/min) vs. temperature of polymers, PN−1 (**A**), PN−05 (**B**), PN−01 (**C**), PN−005 (**D**), obtained at different heating rates at 5, 10, 15 and 20 °C min^−1^ in a nitrogen atmosphere of 25 mL min^−1^.

The thermal degradations of the synthesized four NIPA homopolymers, PN-1, PN-05, PN-01 and PN-005, were studied by determining their mass loss (TG%) during different heating rates (*β*) at 5, 10, 15, and 20 °C min^−1^. Three thermogravimetric measurements were performed for each compound to check the repeatability. All experiments were reproducible with a 5% accuracy. The results of an exemplary series of thermogravimetric experiments (mass loss as a function of temperature) and their derivatives for all polymers tested are shown in Figure 3 and Figure 4, respectively. 

Table 2 summarizes the evaluated thermal decomposition parameters for all samples at different heating rates for the main degradation process: *T_Onset_*—extrapolated onset temperature of decomposition, *T_m_*—temperature of maximum weight loss rate, *T_Endset_*—extrapolated temperature at which the degradation process ended, *T*_0.5wt.%_—the temperature at which 0.5 wt.% loss occurred, *Res* amount of residue at 700 °C.

### 3.5. Kinetic Analysis

Kinetic analysis was carried out for thermal analytical data corresponding to the third, main decomposition process, in an approximate range from 350 °C to 440 °C using all heating rates and the five calculation procedures described by Ozawa, Kissinger, Flynn—Wall—Ozawa (FWO), Kissinger–Akahira–Sunose (KAS) and Friedman (FD). Each point on the graph is the average of three series of experiments conducted for each compound tested: PN-1, PN-05, PN-01 or PN-005. In the FWO, KAS and FD models, kinetic parameters were determined using a temperature corresponding to specific values of the degree of conversion (*α*) in the range of 0.1–0.9 with a 0.1 step and the heating rate applied. In these methods, the activation energy depended on the degree of conversion.

**Figure 5:**
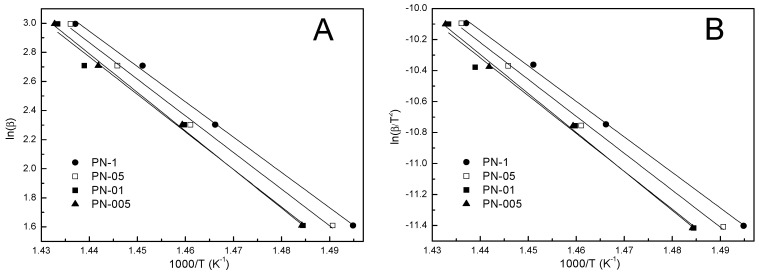
The Ozawa (**A**) and Kissinger (**B**) plots at different heating rates for polymers PN-1, PN-05, PN-01, PN-005.

Using the least-squares linear regression from the Ozawa plot of ln*β* versus 1000/*T* K^−1^ (Figure 5A) and from the Kissinger plot of ln(*β/T*^2^) versus 1000/*T* K^−1^ (Figure 5B), straight lines were obtained with a square of linear correlation coefficient r^2^ close to unity. The activation energy was calculated from the slope of the graphs according to Equations (2) and (3). By using the methods of Ozawa and Kissinger, the obtained values of activation energy were assumed to be constant *E_a_* and were not calculated at progressive *α* values. The results for PN-1, PN-05, PN-01 and PN-005 are shown in Table 3, Table 4, Table 5 and Table 6, respectively. The values of the kinetic parameters determined according to these two methods differed by 6%, and Ozawa equation gave the higher value.

**Figure 6:**
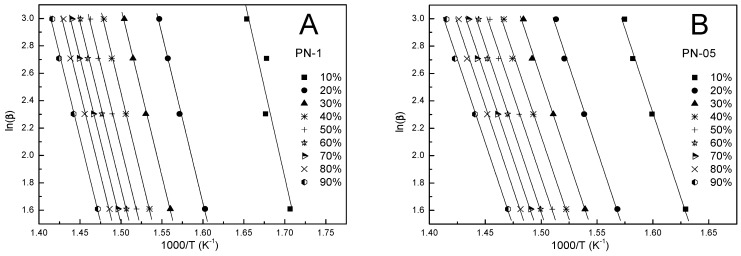

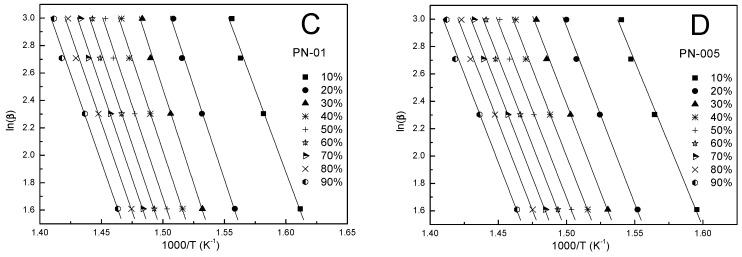
The FWO plots at different heating rates for polymers PN-1 (**A**), PN-05 (**B**), PN-01 (**C**), and PN-005 (**D**).

**Figure 7:**
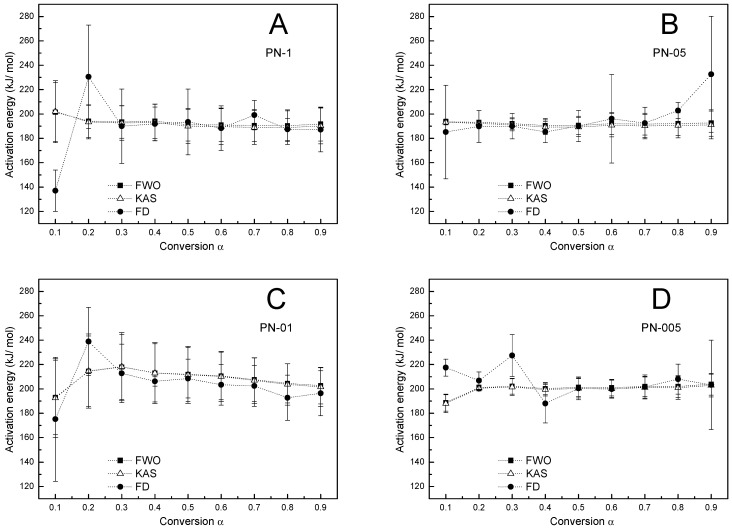
The dependence of activation energy on the conversion (*α*) for the thermal degradation of polymers PN−1 (**A**), PN−05 (**B**), PN−01 (**C**), PN−005 (**D**), according to FWO, KAS and FD methods.

Kinetic parameters obtained by the FWO method were calculated according to Equation (4) for a given conversion value (*α*). Figure 6A–D shows the FWO plots of ln(*β*) against 1000/*T*. The values of the activation energy obtained from the slope were in the ranges 190.4–201.5, 190.6–193.7, 193.0–218.0, 188.8–203.9 kJ/mol for PN-1, PN-05, PN-01, PN-005, respectively, and were summarized in Table 3, Table 4, Table 5 and Table 6. The comparison shows that the obtained *E_a_* values for each tested compound differed depending on the degree of conversion (cf. Figure 7A–D). These differences were not large. The largest discrepancies from the other results occured at an alpha of 0.1 and ranged from 1.5 to 11.5%. The mean values of the activation energy were 193.1, 192.1, 208.5 and 200.3 kJ/mol for PN-1, PN-05, PN-01 and PN-005, respectively (cf. Table 7).

**Figure 8:**
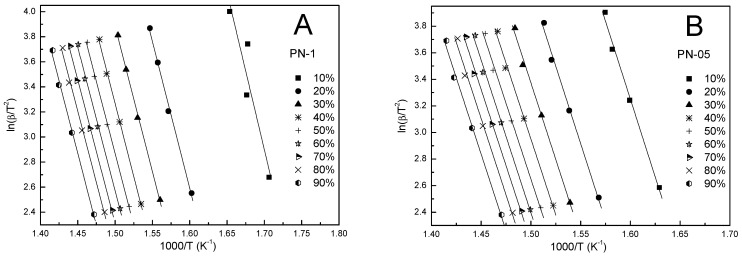

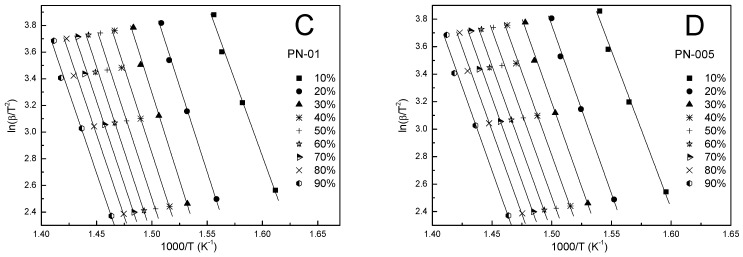
The KAS plots at different heating rates for polymers PN-1 (**A**), PN-05 (**B**), PN-01 (**C**), PN-005 (**D**).

In addition, the KAS method was also used to calculate the kinetic parameters. According to Equation (5), the dependence of ln(*β/T*^2^) on 1000/*T* was determined for the different conversion values (cf. Figure 8A–D). Based on the slope and the intercept of the obtained straight lines, with a correlation coefficient close to unity, the activation energy and pre-exponential factor were calculated, respectively. From Table 3, Table 4, Table 5 and Table 6 and Figure 7A–D, it can be seen that the values of the activation energy varied slightly depended on the degree of conversion and were in the ranges 188.6–202.0, 189.3–193.3, 218.3–192.5, 187.9–202.8 kJ/mol for PN-1, PN-05, PN-01, PN-005, respectively. Over the whole conversion range, except for α equal to 0.1, the values of the apparent activation energy were almost identical. The largest difference in *E_a_* between *α* = 0.1 and the other results occured for PN-01 and was about ~25 kJ/mol. The mean values of the activation energy were 192.1, 190.9, 208.1 and 199.4 kJ/mol for PN-1, PN-05, PN-01 and PN-005, respectively (cf. Table 7).

**Figure 9:**
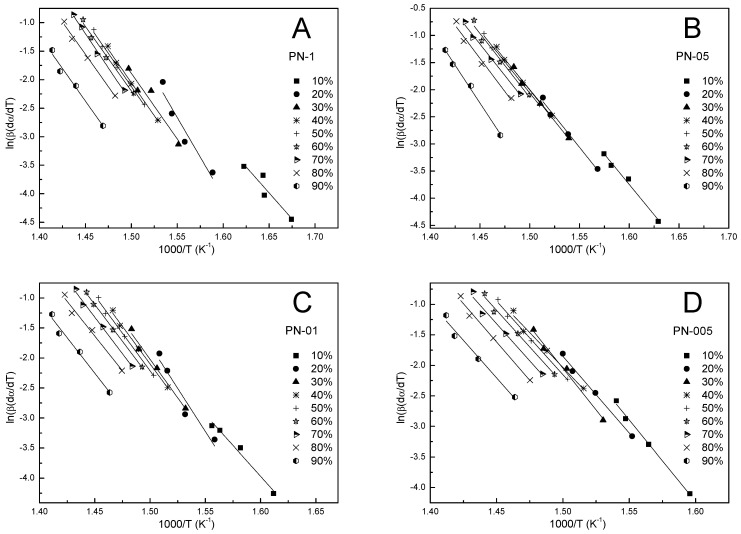
The FD plots at different heating rates for polymers PN-1 (**A**), PN-05 (**B**), PN-01 (**C**), PN-005 (**D**).

The Friedman differential isoconversion (FD) method was used to obtain the kinetic parameters. The activation energy values obtained from plotting ln(*β·dα/dT*) against 1/*T* at each given conversion degree *α* = 0.1–0.9 (cf. Figure 9A–D) for the thermal degradation of PN-1, PN-05, PN-01, PN-005 were in the ranges 137.0–230.5, 185.3–232.6, 175.2–239.0, 188.0–227.4 kJ/mol, respectively. The results obtained are shown in Figure 7A–D and summarized in Table 3, Table 4, Table 5 and Table 6. The dependence of the activation energy on the conversion value showed high variability at the initial stages (*α* = 0.1–0.3) of PN-1, PN-01, PN-005 and the final stages (*α* = 0.8–0.9) for PN-05. The mean values of the activation energies were 189.4, 196.1, 204.1 and 205.1 kJ·mol^−1^ for PN-1, PN-05, PN-01 and PN-005, respectively (cf. Table 7).

## 4. Discussion

### 4.1. XRPD

The diffractograms of the samples showed broad diffraction lines, which could indicate that the sample had an amorphous-crystalline structure. Broad peaks of low-intensity could imply a small crystallite size or a particle size below 100 nm [48,51].

### 4.2. ATR-FTIR

The absence of peaks characteristic of an unsaturated C=C bond in the spectra of all PN-1, PN-05, PN-01 and PN-005 polymers indicated that the vinyl bond was saturated and a polymerization process had occurred, confirming that PN-1, PN-05, PN-01 and PN-005 were in their polymer form.

### 4.3. TGA

The TG-thermograms for all samples showed a similar trend: as the heating rate increased, the thermal degradation profiles (TGA) shifted toward higher temperatures. Similarly, the thermal degradation rate curves (DTG) were found to shift regularly toward higher temperatures as the heating rate increased. It was not noticed that, as the amount of the initiator was reduced, the TG curves in the sequence PN-1, PN-05, PN-01 and PN-001 showed regular shifts toward a higher or lower degradation temperature value. The values of the temperature of a maximum mass loss rate (*T_m_*) at the same heating rates for PN-1, PN-05, PN-01 and PN-001 were almost the same. The differences between them did not exceed 2% (cf. Table 2). The thermal behavior of the four compounds studied were similar to each other, due to the presence of identical chemical bonds in their chemical structures, and were characterized by the occurrence of three mass loss stages, as shown in Figure 4. The first, observed at about 90 °C and accompanied by a mass loss of 5.00–10.00%, could be explained by the evaporation of water molecules bound in the polymers by hydrogen bonds to the NH and C=O groups of NIPA. The second, occurred very close the main thermal events and took place in the approximate temperature range 300–320 °C, which may have corresponded to the degradation of part of the acrylamide groups. The third main, single and significant degradation stage, started at about 350 °C and ended at about 440 °C, reflecting the complete thermal decomposition of the PN-1, PN-05, PN-01 and PN-005 polymer chains and was associated with a 70.00–85.00% mass loss. All polymers used in this study were stable up to approximately 300 °C. The values of the temperature of the maximum weight loss rate (*T_m_*) at the same heating rates for PN-1, PN-05, PN-01 and PN-001 were almost the same. The differences between them did not exceed 2% (cf. Table 2).

The increase in the heating rate resulted in lateral shifts in the temperature of the maximum weight loss rate (*T_m_*) toward higher temperatures (cf. Table 2 and Figure 4A–D). This behavior has been explained in various ways in the literature. For example, by the increasing heating speed, and the differences between the furnace and sample temperatures. Slower heating resulted in a gradual increase in temperature at a larger volume of the test sample and thus, at a given temperature, a greater degree of mass loss compared to a faster heating rate occurred. Another reason for *T_m_* shifts might be the changes in the reaction mechanism when the heating rate was alterated [55,56].

### 4.4. Kinetic

**Figure 10:**
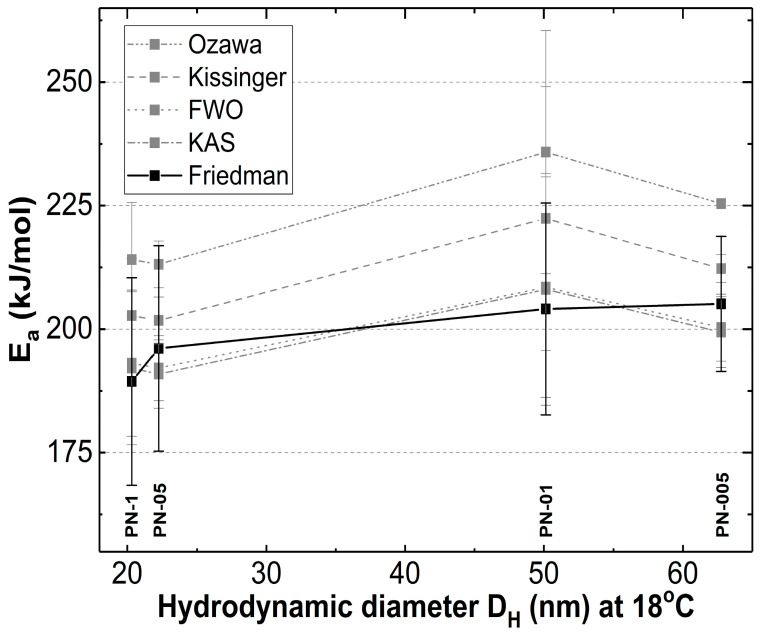
The dependence of activation energy on the particle size at 18 °C for the prepared polymers PN-1, PN-05, PN-01, PN-005.

From the data presented in Table 3, Table 4, Table 5 and Table 6 and in Figure 10, it can be clearly seen that the values of *E_a-α_* obtained by FWO, KAS and FD methods were found practically constant in a 0.1–0.9 conversion range, indicating that thermal degradation was the main process.

According to the graph in Figure 10, *E_a_* increased with the particle size only in the model proposed by Friedmann (Equation (5)) [38]. In the case of Kissinger, Ozawa, FWO and KAS models only partially in agreement with the *Ea* particle size was observed. The Friedmann model, as the most sophisticated, could reflect the effect of the particle size on *E_a_* over the entire range studied. This is probably due to the fact that Friedman activation energies are independent of the heating rate range, which may reduce the systematic error [57,58,59].

From all the results presented, it was difficult to find a linear relationship between the activation energy and the amount of initiator used in the polymerization reaction. The activation energy values obtained by the previously mentioned and described five methods for one type of polymer were in good agreement with each other. However, the activation energies calculated by the Kissinger and Ozawa methods were more similar to each other than the activation energy calculated by the KAS, FWO and FD methods. The analysis of the activation energies, calculated by one method, between the different polymers PN-1, PN-05, PN-01 and PN-005 showed that the activation energies obtained were similar. However, it could be seen that the activation energies obtained for PN-01 and PN-005 were slightly higher than those calculated for the PN-1 and PN-05. The similar values of activation energy might be due to the fact that polymers PN-1, PN-05, PN-01 and PN-005 were composed of the same monomer. The only difference between the polymers tested was the different amounts of initiator used during the synthesis because, as we have shown in our previous studies, the initiator concentration affected the polymer particle size [47]. Particles synthesized at higher initiator concentrations had a low average hydrodynamic diameter (D_H_), potentially indicating shorter polymer chains. This phenomenon may explain the lower activation energy of the polymers PN-1 and PN-05 compared to polymers PN-01 and PN-005. This is also an additional confirmation of our previous studies.

## 5. Conclusions

Four NIPA polymers, PN-1, PN-05, PN-01 and PN-005, were successfully prepared by the SFPP method using different concentrations of the KPS initiator. The polymerization reaction was confirmed via ATR-FTIR analysis through the absence of carbon–carbon double bonds and absorption peaks in the spectra of the synthesized polymers—Figure 2. The diffraction patterns of the polymers studied had a characteristic halo with two broad diffraction peaks at ~8°, and 20° 2θ, indicating a semi-crystalline material. Thermal decompositions of the synthesized materials were carried out in a nitrogen atmosphere at different heating rates of 5, 10, 15 and 20 °C per minute. The analyzed polymers showed good thermal stability up to 300 °C. The thermograms of the polymers studied showed a three-stage mass loss; however, almost total degradation took place in one main process occurring in the temperature range of 395–425 °C. The kinetics of the thermal degradation was determined from thermogravimetric analysis. Kinetic parameters were determined using five isoconversion methods: Kissinger, Ozawa, KAS, FWO and Friedman. The activation energy values obtained with these methods were similar. 

However, differences could be noticed between the activation energy calculated by the Kissinger and Ozawa methods and the KAS, FWO and FD methods. There were also slight differences between activation energies calculated for the PN-1, PN-05 polymers and activation energies for polymers PN-01, PN-005. Planning is a very important issue when carrying out a synthesis, taking into account criteria such as, e.g., efficiency and execution time. Our in-depth study on the subject shows that it is possible to plan or predict already at the synthesis stage to obtain a final product that is characterized by specific and desired physicochemical properties, such as activation energy.

## Figures and Tables

**Table 1:** Composition of investigated preparations.

Type of Nanosphere *	Monomer NIPA [g]	Initiator KPS [g]
PN-1	5.002	5.972
PN-05	5.004	2.986
PN-01	5.006	0.598
PN-005	5.002	0.300

* The acronyms PN-1, PN-05, PN-01, PN-005 reflect the acronyms of macromolecules for the same composition, synthesized in the study [47]: P-1, P-05, P-01, P-005, respectively.

**Table 2:** The characteristic parameters of thermal degradation curves for PN-1, PN-05, PN-01, PN-005.

Sample	Heating Rate [°C min^−1^]	*T_m_* [°C]	Rate of Mass Loss [% min^−1^]	*T_Onset_* [°C]	*T_Endset_* [°C]	*Res* [%]	*T*_0.5wt.%_ [°C]
PN-005	5	400.6	10.69	372.6	394.5	4.79	269.0
	10	412.1	19.55	380.6	427.2	5.63	275.0
	15	420.4	27.51	387.0	436.2	5.42	253.0
	20	424.8	35.97	395.1	440.8	5.23	193.0
PN-01	5	400.5	10.40	369.2	413.1	4.58	260.0
	10	411.9	19.87	380.7	425.4	6.88	272.0
	15	421.8	26.92	387.7	436.2	3.99	239.0
	20	424.5	34.50	394.1	440.8	4.25	201.0
PN-05	5	397.7	11.04	372.8	392.5	4.60	260.0
	10	411.3	19.61	380.7	424.6	6.74	244.0
	15	418.5	28.09	389.1	432.7	4.94	194.0
	20	423.2	34.88	393.8	438.6	5.26	175.0
PN-1	5	395.8	8.92	354.9	417.2	9.43	209.0
	10	408.9	16.44	374.6	423.8	10.07	198.0
	15	416.0	23.95	381.4	432.6	6.44	127.0
	20	422.7	29.0	390.6	437.8	7.81	151.1

**Table 3:** Values of activation energies (*E_a_*), pre-exponential factors (*A*) and regression coefficients for PN-1 obtained by Ozawa, Kissinger, FWO, KAS and FD methods.

		PN-1								
Methods		Conversion, *α*								
		0.10	0.20	0.30	0.40	0.50	0.60	0.70	0.80	0.90
FWO	*E_a_* kJ mol^−1^	201.49 ± 24.21	194.05 ± 13.24	193.49 ± 13.41	194.07 ± 14.15	191.21 ± 13.28	191.12 ± 13.87	190.50 ± 13.24	190.42 ± 13.02	191.81 ± 13.95
	*A*	5.55 × 10^21^	3.89 × 10^18^	8.58 × 10^17^	3.96 × 10^17^	1.31 × 10^17^	1.02 × 10^17^	6.58 × 10^16^	4.82 × 10^16^	4.78 × 10^16^
	r^2^	0.8976	0.9963	0.9987	0.9987	0.9978	0.9971	0.9969	0.9963	0.9962
KAS	*E_a_* kJ mol^−1^	201.99 ± 25.47	193.51 ± 13.95	192.63 ± 14.13	193.05 ± 14.90	189.93 ± 14.00	189.74 ± 14.61	189.00 ± 13.95	188.85 ± 13.72	190.19 ± 14.70
	*A*	2.13 × 10^21^	1.31 × 10^18^	2.74 × 10^17^	1.22 × 10^17^	3.97 × 10^16^	3.02 × 10^16^	1.93 × 10^16^	1.39 × 10^16^	1.35 × 10^16^
	r^2^	0.8887	0.9959	0.9985	0.9986	0.9975	0.9968	0.9966	0.9959	0.9957
Friedman	*E_a_* kJ mol^−1^	137.01 ± 16.97	230.51 ± 42.59	189.98 ± 30.55	191.94 ± 13.90	193.47 ± 26.97	188.39 ± 18.20	199.13 ± 12.04	187.37 ± 9.06	187.16 ± 18.22
	*A*	2.97 × 10^11^	4.49 × 10^20^	2.40 × 10^15^	7.7 × 10^14^	4.73 × 10^15^	6.43 × 10^14^	9.2 × 10^14^	7.25 × 10^13^	6.92 × 10^13^
	r^2^	0.8917	0.9324	0.9367	0.9952	0.9948	0.9906	0.9958	0.9934	0.9812
Kissinger	*E_a_* kJ mol^−1^	202.75 ± 11.61								
	*A*	2.06 × 10^11^								
	r^2^	0.9899								
Ozawa	*E_a_* kJ mol^−1^	214.06 ± 11.60								
	*A*	7.02 × 10^17^								
	r^2^	0.9910								

**Table 4:** Values of activation energies (*E_a_*), pre-exponential factors (*A*) and regression coefficients for PN-05 obtained by Ozawa, Kissinger, FWO, KAS and FD methods.

		PN-05								
Methods		Conversion, *α*								
		0.10	0.20	0.30	0.40	0.50	0.60	0.70	0.80	0.90
FWO	*E_a_* kJ mol^−1^	193.70 ± 2.17	193.15 ± 0.71	192.16 ± 4.69	190.55 ± 5.71	190.67 ± 7.48	192.12 ± 9.04	191.66 ± 8.92	192.19 ± 9.68	192.66 ± 10.84
	*A*	1.12 × 10^18^	2.17 × 10^17^	1.12 × 10^17^	5.91 × 10^16^	5.17 × 10^16^	6.11 × 10^16^	4.39 × 10^16^	4.16 × 10^16^	3.86 × 10^16^
	r^2^	0.9936	0.9945	0.9956	0.9949	0.9950	0.9951	0.9950	0.9940	0.9945
KAS	*E_a_* kJ mol^−1^	193.31 ± 2.27	192.33 ± 0.76	191.07 ± 4.95	189.26 ± 6.02	189.29 ± 7.88	190.74 ± 9.52	190.19 ± 9.39	190.67 ± 10.20	191.08 ± 11.41
	*A*	3.90 × 10^17^	7.00 × 10^16^	3.50 × 10^16^	1.79 × 10^16^	1.54 × 10^16^	1.79 × 10^16^	1.27 × 10^16^	1.19 × 10^16^	1.09 × 10^16^
	r^2^	0.9929	0.9939	0.9951	0.9943	0.9946	0.9945	0.9944	0.9933	0.9939
Friedman	*E_a_* kJ mol^−1^	185.26 ± 38.41	189.83 ± 13.17	190.02 ± 10.42	185.28 ± 8.85	190.16 ± 12.74	196.12 ± 36.42	192.53 ± 12.99	202.90 ± 6.64	232.59 ± 47.57
	*A*	1.03 × 10^17^	6.03 × 10^14^	2.35 × 10^14^	7.73 × 10^13^	2.72 × 10^14^	6.52 × 10^16^	4.31 × 10^14^	8.43 × 10^14^	5.96 × 10^19^
	r^2^	0.9881	0.9879	0.9907	0.9960	0.9955	0.9773	0.9926	0.9799	0.9954
Kissinger	*E_a_* kJ mol^−1^	201.76 ± 4.74								
	*A*	7.21 × 10^10^								
	r^2^	0.9926								
Ozawa	*E_a_* kJ mol^−1^	213.11 ± 4.73								
	*A*	2.48 × 10^17^								
	r^2^	0.9933								

**Table 5:** Values of activation energies (*E_a_*), pre-exponential factors (*A*) and regression coefficients for PN-01 obtained by Ozawa, Kissinger, FWO, KAS and FD methods.

		PN-01								
Methods		Conversion, *α*								
		0.10	0.20	0.30	0.40	0.50	0.60	0.70	0.80	0.90
FWO	*E_a_* kJ mol^−1^	193.03 ± 30.66	214.48 ± 28.95	218.03 ± 26.60	213.15 ± 23.87	211.92 ± 22.19	210.63 ± 19.57	207.61 ± 17.77	204.59 ± 16.14	202.68 ± 15.03
	*A*	1.65 × 10^20^	1.76 × 10^21^	9.10× 10^20^	1.17 × 10^20^	3.99 × 10^19^	1.26 × 10^19^	3.95 × 10^18^	1.02 × 10^18^	4.18 × 10^17^
	r^2^	0.9935	0.9955	0.9947	0.9935	0.9927	0.9931	0.9930	0.9929	0.9928
KAS	*E_a_* kJ mol^−1^	192.49 ± 32.23	214.71 ± 30.43	218.26 ± 27.97	213.00 ± 25.10	211.61 ± 23.33	210.17 ± 20.58	206.92 ± 18.69	203.67 ± 16.98	201.57 ± 15.81
	*A*	5.59 × 10^19^	5.59 × 10^20^	2.80 × 10^20^	3.5 × 10^19^	1.18 × 10^19^	3.67 × 10^18^	1.14 × 10^18^	2.88 × 10^17^	1.17 × 10^17^
	r^2^	0.9928	0.9950	0.9942	0.9928	0.9919	0.9924	0.9923	0.9921	0.9919
Friedman	*E_a_* kJ mol^−1^	175.17 ± 50.77	238.91 ± 28.01	212.82 ± 23.94	206.19 ± 3.83	208.49 ± 15.94	203.43 ± 16.74	202.50 ± 16.73	192.75 ± 18.60	196.44 ± 18.47
	*A*	5.11 × 10^16^	8.94 × 10^19^	2.85 × 10^17^	2.02 × 10^15^	8.36 × 10^15^	4.01 × 10^15^	2.3 × 10^15^	4.46 × 10^14^	4.77 × 10^14^
	r^2^	0.9799	0.9378	0.9845	0.9945	0.9929	0.9981	0.9934	0.9825	0.9828
Kissinger	*E_a_* kJ mol^−1^	222.41 ± 26.68								
	*A*	1.20 × 10^14^								
	r^2^	0.9853								
Ozawa	*E_a_* kJ mol^−1^	235.84 ± 24.62								
	*A*	4.19 × 10^20^								
	r^2^	0.9900								

**Table 6:** Values of activation energies (*E_a_*), pre-exponential factors (*A*) and regression coefficients for PN-005 obtained by Ozawa, Kissinger, FWO, KAS and FD methods.

		PN-005								
Methods		Conversion, *α*								
		0.10	0.20	0.30	0.40	0.50	0.60	0.70	0.80	0.90
FWO	*E_a_* kJ mol^−1^	188.80 ± 6.98	201.07 ± 2.19	202.16 ± 6.51	200.30 ± 5.03	201.36 ± 7.57	200.98 ± 6.81	202.08 ± 8.22	202.00 ± 8.95	203.87 ± 8.91
	*A*	3.10 × 10^17^	7.37 × 10^17^	7.82 × 10^17^	3.10 × 10^17^	3.70 × 10^17^	2.31 × 10^17^	2.58 × 10^17^	2.18 × 10^17^	2.16 × 10^17^
	r^2^	0.9917	0.9953	0.9960	0.9954	0.9950	0.9942	0.9940	0.9934	0.9924
KAS	*E_a_* kJ mol^−1^	187.94 ± 7.32	200.55 ± 2.32	201.54 ± 6.85	199.47 ± 5.30	200.49 ± 7.97	200.02 ± 7.17	201.11 ± 8.66	200.95 ± 9.42	202.83 ± 9.38
	*A*	1.03 × 10^17^	2.32 × 10^17^	2.40 × 10^17^	9.33 × 10^16^	1.09 × 10^17^	6.75 × 10^16^	7.44 × 10^16^	6.21 × 10^16^	6.06 × 10^16^
	r^2^	0.9908	0.9950	0.9956	0.9950	0.9943	0.9936	0.9934	0.9927	0.9916
Friedman	*E_a_* kJ mol^−1^	217.46 ± 6.93	206.81 ± 7.16	227.40 ± 17.25	188.00 ± 15.80	200.52 ± 9.37	199.87 ± 7.48	201.58 ± 9.89	208.03 ± 12.31	203.34 ± 36.58
	*A*	3.63 × 10^16^	4.51 × 10^15^	4.42 × 10^17^	2.48 × 10^14^	1.29 × 10^15^	8.41 × 10^14^	9.75 × 10^14^	3.22 × 10^15^	1.7 × 10^16^
	r^2^	0.9954	0.9944	0.9893	0.9893	0.9936	0.9910	0.9801	0.9879	0.9816
Kissinger	*E_a_* kJ mol^−1^	212.25 ± 2.80								
	*A*	3.45 × 10^11^								
	r^2^	0.9899								
Ozawa	*E_a_* kJ mol^−1^	225.37 ± 0.49								
	*A*	1.51 × 10^18^								
	r^2^	0.9857								

**Table 7:** Comparison of the mean values of activation energies (*E_a_*), pre-exponential factors (*A*) and regression coefficients obtained for PN-1, PN-05, PN-01, PN-005 by Ozawa, Kissinger, FWO, KAS and FD methods.

		Ozawa	Kissinger	FWO	KAS	FD
PN-1	*E_a_* kJ mol^−1^	214.06 ± 11.60	202.75 ± 11.61	193.10 ± 14.80	192.10 ± 15.50	189.40 ± 21.00
	*A*	7.02 × 10^17^	2.06 × 10^11^	6.18 × 10^20^	2.36 × 10^20^	4.98 × 10^19^
	r^2^	0.9910	0.9899	0.9862	0.9849	0.9680
PN-05	*E_a_* kJ mol^−1^	213.11 ± 4.73	201.76 ± 4.74	192.10 ± 6.60	190.90 ± 6.93	196.10 ± 20.8
	*A*	2.48 × 10^17^	7.21 × 10^10^	1.97 × 10^17^	6.50 × 10^16^	6.64 × 10^18^
	r^2^	0.9933	0.9926	0.9947	0.9941	0.9892
PN-01	*E_a_* kJ mol^−1^	235.84 ± 24.62	222.41 ± 26.68	208.50 ± 22.31	208.05 ± 23.46	204.08 ± 21.45
	*A*	4.19 × 10^20^	1.20 × 10^14^	3.35 × 10^20^	1.05 × 10^20^	9.97 × 10^18^
	r^2^	0.9900	0.9853	0.9935	0.9928	0.9829
PN-005	*E_a_* kJ mol^−1^	225.40 ± 0.49	212.25 ± 2.80	200.3 ± 6.78	199.40 ± 7.16	205.10 ± 13.65
	*A*	1.51 × 10 ^18^	3.45 × 10^11^	3.82 × 10^17^	1.16 × 10^17^	5.63 × 10^16^
	r^2^	0.9857	0.9899	0.9942	0.9899	0.9893

## Data Availability

The data are available in the Department of Physical Chemistry and Biophysics, Pharmaceutical Faculty, Wroclaw Medical University.
